# Acute Pancreatitis Review

**DOI:** 10.5152/tjg.2023.23175

**Published:** 2023-08-01

**Authors:** Yuting Huang, Dilhana S. Badurdeen

**Affiliations:** Department of Gastroenterology and Hepatology, Mayo Clinic, FL, USA

**Keywords:** Acute pancreatitis, management, fluid resuscitation, pain management, nutrition support

## Abstract

Acute pancreatitis, a prevalent illness with devastating consequences, poses a grave threat to those affected. There has been a steady increase in the occurrence of acute pancreatitis at about 3% per year from 1961 to 2016. There are 3 main guidelines on acute pancreatitis, including the American College of Gastroenterology, the International Association of Pancreatology/American Pancreatic Association guideline in 2013, and the American Gastroenterological Association guideline in 2018. However, several milestone studies have been published since then. We hereby reviewed the current acute pancreatitis guidelines with an update on clinical practice-changing literature. The aggressive or moderate fluid resuscitation in acute pancreatitis (WATERFALL) trial recommended fluid resuscitation with lactated Ringer’s solution at a moderate aggressive rate. All guidelines did not recommend prophylactic antibiotics use. Early enteral feeding reduces morbidity. A clear liquid diet is no longer recommended. Nutrition with nasogastric or nasojejunal feeding does not have a difference. The upcoming high vs. low-energy administration in the early phase of acute pancreatitis (GOULASH) trial will provide more information on the impact of calorie intake. Pain management should be individualized based on the degree of pain and severity of pancreatitis. In patients with moderate to severe and severe acute pancreatitis, a step-down approach with epidural analgesia can be considered for moderate to severe pain. The management of acute pancreatitis has evolved. New research on the impact of electrolytes, pharmacologic agents, the role of anticoagulants, and nutrition support will provide scientific and clinical evidence to improve patient care and decrease morbidity and mortality.

**Main Points**

- The management of pancreatitis has evolved rapidly in the last decade, with several milestone studies that have been published since the last guideline.
- Moderate-aggressive fluid resuscitation with lactated Ringer’s, early enteral feeding with a low-fat diet, no prophylactic antibiotics, and an individualized pain management plan is recommended in acute pancreatitis.
- New research on the impact of electrolytes, pharmacologic agents, the role of anticoagulants, and nutrition support will be promising to improve the treatment of acute pancreatitis in the future.

## Introduction

If not properly managed, the morbidity and mortality of acute pancreatitis can be significant. Severe acute pancreatitis can lead to serious complications and can be life threatening, with a mortality rate of 10%-30%. Acute pancreatitis is a prominent gastrointestinal (GI) reason for hospitalization in the United States, resulting in roughly 300 000 visits to the emergency department annually.^1^ There has been a steady increase in the occurrence of acute pancreatitis at about 3% per year from 1961 to 2016, in North America and Europe.^2^ Increased prevalence of risk factors (alcohol consumption, obesity, and high-fat diet) improved diagnostic techniques, longevity, and disease awareness might all contribute to the rising incidence. Prompt initiation of treatment is essential in reducing mortality rates and minimizing the risk of infectious complications based on the 3 published guidelines; The American College of Gastroenterology (ACG) guideline: management of acute pancreatitis^3^ published in 2013, Association of Pancreatology (IAP)/American Pancreatic Association (APA) evidence-based guidelines for the management of acute pancreatitis^4^ published in 2013, and American Gastroenterological Association (AGA) Institute Guideline on initial management of acute pancreatitis^5^ published in 2018. However, the treatment of acute pancreatitis has evolved due to several key publications including strong evidence-based randomized control trials (RCTs), which may impact clinical practice profoundly. In this review, we will summarize the current guidelines and discuss recent changes in the treatment of acute pancreatitis, areas of ongoing controversy, and future direction.

## Clinical and Research Consequences

The original 1992 Atlanta classification of acute pancreatitis^6^ established a uniform system for reporting research findings and facilitated communications among healthcare providers. According to the 2012 revision of the Atlanta classification,^7^ moderately severe acute pancreatitis is characterized by transient organ failure, local complications, or exacerbation of comorbidities. Severe acute pancreatitis, on the other hand, is defined by persistent organ failure lasting more than 48 hours. Early recognition and treatment of severe pancreatitis are crucial in reducing morbidity and mortality rates, as severe acute pancreatitis has a significantly higher mortality rate of 15%-30% compared to the 0%-1% mortality rate associated with mild acute pancreatitis.^8^ Multiple methods of stratification have been created to facilitate the timely identification of severe acute pancreatitis. These include the sequential organ failure assessment, bedside index for severity of acute pancreatitis, Ranson criteria, and acute physiology and chronic health evaluation II. Local complications include peripancreatic fluid collections, sterile or infected pancreatic and peripancreatic necrosis, pseudocyst, and sterile or infected walled-off necrosis.

## Intravenous Fluid Resuscitation

Many negative predictive aspects of acute pancreatitis stems from significant loss of fluid into the third space and depletion of intravascular volume.^9^ It was believed that early and aggressive fluid resuscitation is associated with lower rates of mortality.^1^ The ACG guideline recommended aggressive hydration, with isotonic crystalized solution at 250-500 mL per hour in the absence of cardiovascular, or renal disease. The guideline also highlighted that aggressive hydration in the early stage of acute pancreatitis is particularly advantageous within the first 12-24 hours, but may offer limited benefits beyond this time frame.^3^ According to the IAP/APA guideline, the recommended approach is to administer intravenous (i.v.) fluid in a goal-directed manner, with an initial rate of 5-10 mL/kg/h, until resuscitation objectives have been reached.^4^ Parameters for goal-directed management includes urine output more than 0.5-1 mL/kg/h, hematocrit at 35%-44%, heart rate (HR) less than 120 bpm, mean arterial pressure in the range 65-85 mmHg, and normal blood urea nitrogen level (BUN). Central venous pressure (CVP) can be measured to avoid volume overload, and an increase in CVP between 2 to 5 mmHg indicates fluid resuscitation is efficient and sufficient. If CVP increase less than 2 mmHg, the fluid challenge is efficient but not sufficient, and if CVP increase more than 5 mmHg indicates fluid infusion should be held for a while before restarting again. The AGA guideline recommended goal-directed therapy for fluid management, albeit with evidence of very low quality.^5^ Notably, the AGA technical review demonstrated that goal-directed therapy did not yield a significant improvement in mortality rates, prevention of pancreatic necrosis, or reduction in the incidence of persistent multiple organ failure when compared to nontargeted therapy.^10^

A meta-analysis in 2020 raised the concern that administering i.v. fluids aggressively in the early stages did not show any improvement in mortality rates. On the contrary, it could potentially elevate the risk of acute kidney injury and pulmonary edema, resulting in respiratory failure and the need for mechanical ventilation. *New England Journal of Medicine* published the WATERFALL trial in September 2022, which provided new evidence supporting moderate fluid resuscitation. In this RCT of 249 patients from 18 centers, aggressive fluid resuscitation (3 mL/kg/h after a bolus of 20 mL/kg) was compared to moderate fluid resuscitation (in patients with hypovolemia, a 10 mL/kg bolus is administered, whereas those with euvolemia do not receive the bolus). Following this, all patients are given a continuous infusion of 1.5 mL/kg/h with lactated Ringer’s (LR) solution. The trial was halted because although there was no significant difference in the occurrence of moderately severe or severe pancreatitis between the groups, the patients who underwent aggressive resuscitation had a fluid overload rate of 20.5%, which was significantly higher than 6.3% in patients who received moderate resuscitation.^11^ In addition, during the 72-hour time period, the aggressive-resuscitation group received a higher total volume of fluid (8.3 L) comparing to the moderate-resuscitation group (6.6 L), implying that the infusion rate should not only be slower but also the total infused volume should be reduced.^11^ This finding highlights the importance of limiting the overall amount of fluid administered. Monitoring patient’s clinical and hemodynamic status diligently during the initial 72-hour period after admission is crucial to maintain their euvolemia and prevent fluid overload. Administering diuretics to patients who experience fluid overload within the first 72 hours is highly likely to be beneficial and does not adversely affect clinical outcomes.^12^

Both ACG and IAP/APA guidelines recommended LR over normal saline (NS).^3,4^ American Gastroenterological Association makes no recommendation as to whether NS or LR should be used; however, it does not recommend the use of hydroxyethyl starch (HES) fluids.^5^ Theoretically, LR is more pH balanced, while normal saline’s pH is about 5.5. Experimental studies have shown that a low pH level triggers trypsinogen activation, rendering acinar cells more vulnerable to injury and thereby aggravating the severity of established acute pancreatitis.^13^ In 2011, an RCT conducted at 3 sites and on 40 patients showed that there was a significant reduction in systematic inflammation response syndrome (SIRS) and the levels of C-reactive protein (CRP) were lowered after 24 hours in patients resuscitated with LR, compared with NS.^13^ In 2021, a double-blinded RCT with 121 patients compared resuscitation with LR vs. Normal saline 3 mL/kg/h after a 10 mL/kg bolus. There was no difference in the prevalence of SIRS, but LR shortened length of stay (LOS) and decreased intensive care unit (ICU) admission.^14^ Meta-analysis and systematic review summarizing 2 cohort studies and 4 RCTs on this topic with 549 patients (230 in LR group and 319 in NS group) showed no significant difference in mortality and SIRS at 24 hours. Overall, ICU admission and LOS are lower in the LR group.^15^

Several studies have focused on the significance of maintaining phosphate balance during fluid resuscitation for acute pancreatitis. An animal study in 2021 by Farooq et al^16^ showed that administering ethanol intragastrically to mice that were on a low-phosphate diet resulted in severe pancreatitis. However, phosphate repletion helped alleviate the condition. Phosphate supplementation prevented cellular injury caused by ethanol. It has also been shown that severe alcoholic pancreatitis associated with a higher incidence of hypophosphatemia, which has been linked to worse prognosis in a recent retrospective study.^17^ On the other hand, another retrospective clinical study showed that among patients with acute pancreatitis who required ICU admission, those with a high serum phosphate level are more likely to experience mortality.^18^ Individuals with alcoholism have an increased likelihood of developing low levels of serum phosphate, due to baseline malnutrition and are prone to re-feeding syndrome which can also cause hypophosphatemia. Future studies are required to evaluate whether phosphorous supplementation can improve the clinical course of this subgroup of patients with hypophosphatemia.

## Prophylactic Antibiotic Treatment

There is a consensus that all 3 guidelines recommended against prophylactic antibiotic use for acute pancreatitis.^3- 5^ The ACG guideline recommended antibiotics for an extrapancreatic infection,^3^ while the AGA guideline emphasized that prophylactic antibiotics are still not recommended^5^ in necrotizing pancreatitis and severe acute pancreatitis. Despite this consensus, a 2022 systematic review of 21 RCTs with 1383 patients showed that more patients still received prophylactic antibiotics (703 patients received antibiotics while 680 did not). Prophylactic antibiotic administration reduced extrapancreatic infections including sepsis and urinary tract infections; however, the mortality between the 2 groups was similar. Additionally, there was no significant reduction in infected pancreatic necrosis, single-organ failure, or multiorgan failure.^19^

A recent single-center, patient-blinded, RCT showed that a procalcitonin-based algorithm can guide and reduce antibiotic use in patients with acute pancreatitis (PROCAP trial).^20^ The recommendation was to discontinue or refrain from initiating antibiotics when the procalcitonin test result was below 1.0 ng/mL and to commence or maintain antibiotic treatment when the result was 1.0 ng/mL or higher. The utilization of procalcitonin-guided care in patients with acute pancreatitis resulted in a reduction of antibiotic usage without any associated increase in infections or adverse effects. Therefore, the implementation of procalcitonin-based algorithms for the management of this patient population should be considered and potentially integrated into forthcoming guidelines.^21^

## Nutrition Support

### When to Feed?

Historically, patients with acute pancreatitis were kept nothing per os (NPO) to prevent the theoretical possibility of exacerbating inflammation of the pancreas. The Python study^22^ is a landmark study comparing early enteral nutrition with a nasoenteric tube (<24 hours) vs delayed nutrition (>72 hours). This prospective RCT included only patients with predicted severe acute pancreatitis. In patients with acute pancreatitis at high risk for complications, nasoenteric tube feeding initiated early did not demonstrate superiority over an oral diet after 72 hours in decreasing the incidence of infection or mortality.^23^

Over the past 10 years, the perspective on early feeding in acute pancreatitis has undergone a significant shift. Accumulating evidence has indicated that early feeding does not worsen the inflammation of the pancreatic parenchyma and can, in fact, be advantageous for patients with acute pancreatitis.^24^ The ACG guideline recommended oral feeding immediately in mild acute pancreatitis, when patients have no nausea or vomiting, and abdominal pain has resolved.^3^ The IAP/APA guideline recommended oral feeding when inflammatory markers and abdominal pain start showing improvement in predicted acute mild pancreatitis patients. The recommendation from the most recent AGA guideline in 2018 is to initiate oral feeding as tolerated within the first 24 hours instead of maintaining the patient on NPO status.^5^ In the AGA technical review, 11 RCTs were analyzed, and it was determined that there was no significant difference in mortality between early and late feeding. However, delayed feeding was associated with a 2.5-fold higher risk of interventions for necrosis. There was a nonsignificant trend for a higher incidence of total necrotizing pancreatitis, infected peripancreatic necrosis, and multiorgan failure with delayed feeding.^10^ A recent meta-analysis and systematic review summarized 8 RCTs with 748 patients and showed no difference in mortality between early and delayed feeding. Additionally, the early feeding group had a shorter LOS, and there was no difference in the rate of pain relapse, feeding intolerance, progression of acute pancreatitis, or rate of overall complications.^25^ This evidence can be helpful to educate patients who avoid oral intake due to fear of pain.

### How to Feed?

The 3 guidelines concurred on the preference for enteral nutrition over parenteral nutrition.^3- 5^ In the AGA technical review, 12 RCTs were analyzed to compare the use of parenteral feeding to enteral feeding in patients with acute pancreatitis. Patients who receive enteral feeding are associated with a lower risk of infected peripancreatic necrosis, single-organ failure, and multiorgan failure.^10^

Traditionally, enteral nutrition was delivered through a nasojejunal feeding tube, to avoid perceived pancreatic stimulation from gastric feeding.^26^ Nonetheless, all 3 guidelines proposed that enteral nutrition for acute pancreatitis can be delivered through either the nasogastric (NG) or nasojejunal (NJ) route.^3- 5^ The AGA technical review summarized 3 RCTs showing no significant difference in mortality, infected pancreatic necrosis, single-organ failure, multiorgan failure, necrotizing pancreatitis, interventions for necrosis, and ICU need between patients fed with NJ vs. NG tube.^10^ Of note, a more recent systematic review of 5 RCTs with 220 patients showed no difference in mortality, organ failure, infection rate, complications associated with feeds, and exacerbation of pain between patients fed with NJ vs. NG tube.^27^

### What to Feed?

The ACG guideline suggested that a low-fat, solid diet can be initiated for mild acute pancreatitis as it is equally safe compared to a clear liquid diet.^3^ The IAP/APA guideline recommended either polymeric enteral or elemental nutrition formulations in acute pancreatitis,^4^ and the most recent AGA guideline advises for early feeding using a range of diets, such as low fat, normal fat, soft, or solid consistency; therefore, beginning with a clear liquid diet is not deemed necessary.^5^

It has been revealed that both acinar and ductal cells experience ATP depletion subsequent to mitochondrial damage caused cell injury and progression of acute pancreatitis.^28^ Notably, basic science research demonstrated that replenishing ATP levels within cells reinstated their normal functions and safeguarded them from cell death, indicating that restoring the energy level of the pancreas can be beneficial.^29^ However, no clinical study has provided evidence that energy supplementation in acute pancreatitis patients is beneficial. The GOULASH trial is a recent double-blind multicenter RCT comparing low- vs. high-energy administration in acute pancreatitis. Patients in the high-energy group got 30 kcal/kg/day nutrition within the first 24 hours, while the low-energy group was kept NPO on day 0 and day 1 and was started on 10 kcal/kg/day on the second day and gradually increased to 30 kcal/kg/day on day 4. The study compares multiorgan failure lasting more than 48 hours and death as the primary endpoint, and the secondary endpoints included LOS and pain control.^30,31^ Another recent clinical trial to be mentioned is the EFFORT trial studying the effect of dietary fat content’s impact on the recurrence of pancreatitis.^32^ This multicenter RCT compares reduced fat diet vs standard healthy diet on the recurrence rate of acute pancreatitis, the incidence of chronic pancreatitis, and all-cause mortality.^32^ We are eagerly anticipating the results of these ongoing clinical trials, which may potentially provide evidence that changes current clinical practice.

## Pain Management

Abdominal pain is one of the presenting symptoms in patients with acute pancreatitis. Pain management can be clinically challenging; however, there are no guidelines to best manage the treatment of pain in acute pancreatitis. A meta-analysis and systematic review in 2022 of 12 RCTs with 542 patients outlined the pain treatment of acute pancreatitis and demonstrated that epidural analgesia seems to offer the most significant pain relief within the initial 24-hour period, but its efficacy is comparable to opiates after 24 hours. Moreover, NSAIDs provided pain relief similar to opiates within 24 hours.^33^

The mainstay of managing pain consists of opioids and nonsteroidal anti-inflammatory drugs (NSAIDs) currently. When the severity of pain is high, opioids are often employed. A 2019 retrospective study showed that 80% of patients experiencing acute pancreatitis were administered opioids in the early stages of disease.^34^ However, patients treated with opioids often encounter adverse effects such as addiction, sedation, respiratory depression, and opioid-induced bowel dysfunction. This condition is characterized by decreased fluid secretion, increased sphincter tone, and dysmotility, which lead to symptoms such as nausea, vomiting, reflux, bloating, and constipation.^35^ Furthermore, a recent systematic review of RCTs investigating the management of various acute pain conditions has indicated that opioids are not more effective than NSAIDs and, in certain scenarios, may even be less effective.^36^

Thoracic epidural analgesia has been shown to achieve adequate pain control in 87.5% to 100% of patients.^37^ Epidural analgesia may offer a potential advantage in patients with acute pancreatitis as it can enhance pancreatic perfusion, which in turn, may alleviate ischemic injury and inflammation.^38^ Two small RCTs evaluated the safety and efficacy of epidural analgesia in patients with acute pancreatitis. Although they were underpowered, these studies indicated an enhancement in pancreatic perfusion and more effective pain relief within the initial 24 hours^39^ and significantly decreased serum procalcitonin levels.^40^ These encouraging results justified future high-quality and well-powered clinical trials.

To reduce the use of opioids, adjuvant analgesics, particularly neuromodulators like gabapentin and pregabalin, are commonly employed. Several alternative analgesics are under investigation for pain management in acute pancreatitis, including *N*-methyl-d-aspartate (NMDA) receptor blocker, magnesium, ketamine, i.v. lidocaine, and cannabinoids.

The management of pain associated with acute pancreatitis ought to be customized according to the specific needs of each patient. When deciding on the analgesic approach, it is essential to take into account both the severity of pain and the severity of acute pancreatitis. In mild acute pancreatitis patients with mild pain, initial pain management with NSAIDs is suitable. As pain worsens, it may be necessary to escalate to opioids. For patients experiencing moderate to severe pain and diagnosed with moderate to severe acute pancreatitis, a step-down strategy involving epidural analgesia might a suitable option in specific cases, particularly those with underlying cardiorespiratory comorbidities.

## Future Directions

### Systematic Anticoagulation in Severe Acute Pancreatitis

Acute pancreatitis is a procoagulant state. The leakage of activated digestive enzymes from damaged pancreatic acinar cells results in injury to the adjacent vascular epithelial cells, leading to the exposure of tissue factor, activation of platelets, and the initiation of the coagulation cascade. There are animal studies showing that administering warfarin before inducing acute pancreatitis in rats attenuates the development of ischemia/ reperfusion.^41^ The role of anticoagulation in acute pancreatitis is further supported by clinical data. A case–control, retrospective database study showed that patients diagnosed with acute pancreatitis who were under anticoagulation therapy displayed a relatively lower probability of requiring admission to the ICU, encountering acute kidney injury, developing organ failure, or experiencing inpatient mortality. Nonetheless, the group that received anticoagulation had extended hospital LOS and increased hospital expenses.^42^ The group of patients who received anticoagulation showed a significantly greater rate of vessel recanalization. Nevertheless, there was no notable difference in the relative risk of collateral formation, bleeding complications, or death between those who received anticoagulation treatment vs. those that did not.^43^ A meta-analysis in 2019 summarized 16 RCTs on the efficacy of low-molecular-weight heparin (LMWH) in severe acute pancreatitis, which showed patients receiving LMWH treatment had shorter hospital stay, lower mortality, lower incidences of multiorgan failure, pancreatic pseudocyst, and operation rate.^44^ Based on these results, additional research is necessary to acquire a more comprehensive understanding of the potential therapeutic benefits of anticoagulants in the treatment of acute pancreatitis.

## Acute Pancreatitis During the Coronavirus Disease Pandemic

The clinical course of patients infected by the COVID-19 virus can be complicated by acute pancreatitis. However, it is unclear if COVID has a causal relationship with acute pancreatitis.^45^ A meta-analysis in 2022 showed that among 36496 patients, the overall prevalence and mortality for elevated pancreatic enzymes (amylase, lipase, or both, >3 upper limits of normal) were 6.1% and 39.2%, respectively. These patients had worse clinical outcomes, including requirement of ICU admission, mechanical ventilation, and higher mortality.^46^ Another study showed that the pooled mortality was 18.5% in patients with acute pancreatitis coinfected with COVID-19.^47^ The shocking high mortality rate in this patient population warrants future studies on the pathophysiology of COVID-19 causing pancreatic damage and clinical treatment strategies.

## Promising Pharmacologic Agents

There is currently no medication specifically designed for the treatment of acute pancreatitis. A recent meta-analysis and systematic review summarizing 9 studies with 1037 patients showed that combination therapy with somatostatin and ulinastatin (protease inhibitor) significantly reduced the complication rates for acute kidney injury, acute respiratory distress syndrome, and multiorgan dysfunction; however, mortality did not improve.^48^ Furthermore, in an RCT of 79 patients with severe acute pancreatitis, continuous regional arterial infusion (CRAI) of nafamostat, another protease inhibitor, reduced mortality and the need for surgical interventions in comparison to placebo.^49^ In its phase 2 trial, CRAI with nafamostat did not inhibit the development of pancreatic necrosis but showed some early analgesic effect.^50^ Despite the lack of pharmacologic treatments that have been shown to effectively alter the course of acute pancreatitis, there are potential new targets that may offer promise in the future.

## Conclusion

Acute pancreatitis is a prevalent medical condition that carries a substantial risk of morbidity and mortality. Management has evolved rapidly in the last decade, despite published AGA guidelines in 2018. We hereby recommend moderate-aggressive fluid resuscitation with LR in acute pancreatitis, early enteral feeding with a low-fat diet, no prophylactic antibiotics, and an individualized pain management strategy based on the severity of pain and severity of pancreatitis (Supplementary Table 1).

## Figures and Tables

**Supplementary Table 1.** Take-Home Points

**Fluid resuscitation**	**Moderate fluid resuscitation**	Bolus 10 mL/kg only if parent has hypovolemia, followed by 1.5 mL/kg/h.	Infusion stop after 20 hours if oral feeding tolerated >8 hours.	Decrease or stop fluid if suspect volume overload.	*No difference in developing moderate/severe pancreatitis, less incident for volume overload.*	**LR is preferred to NS**	*No difference in developing moderate/severe pancreatitis, organ failure, SIRS. Less LOS and ICU admission.*	**Prophylactic antibiotics**	**No prophylactic antibiotics**	*No difference in mortality, infected pancreatic necrosis, single-organ failure, or multiorgan failure.*	**Nutrition support**	**Recommend early feeding**	*No difference in mortality. Less costs and shorter LOS. No difference in pain relapse rate, feeding tolerance rate, acute pancreatitis progression rate, and overall complications rate.*	*2.5-fold higher risk for intervention for necrosis in delayed feeding group.*	**Recommend enteral feeding**	*Less infected peripancreatic necrosis, single-organ failure and multiorgan failure.*	**Nasogastric tube** ** vs. nasojejunal tube ** **does not matter**	*No difference in mortality, ICU admission, organ failure, rate of infection, complications associated with feeds, and exacerbation of pain.*	**A clear liquid diet is no longer recommended. Instead patients can trial a low-fat regular diet**

LR, lactated Ringer’s; NS, normal saline.
